# Scrub typhus-experience from a South Indian tertiary care hospital

**DOI:** 10.1186/1471-2334-12-S1-P77

**Published:** 2012-05-04

**Authors:** MVS Subbalaxmi, Naval Chandra, VD Teja, Vemu Lakshmi, MN Rao, YSN Raju

**Affiliations:** 1 Department of General Medicine, Nizam’s Institute of Medical Sciences, Hyderabad, India; 2 Department of Microbiology, Nizam’s Institute of Medical Sciences, Hyderabad, India

## Background

Scrub typhus, an emerging rickettsial disease caused by Orientia tsutsugamushi, is spread by the bite of larval trombiculid mites. The infection manifests as a febrile illness with diverse manifestations. This infection has to be differentiated from other tropical fevers. Our experience of 48 patients with scrub typhus admitted recently to our hospital is discussed.

## Methods

Clinical features of 48 patients hospitalized between August and November 2011 with fever, positive OX-K Weil Felix test and clinically diagnosed as scrub typhus were analyzed. Patients with other established causes of fever were excluded.

## Results

All these adult patients hailed from the rural Telangana region of Andhra Pradesh. Eighteen out of 48 were farmers. The average duration of fever was 11days. Eschar was noted in only 12.5% patients. Cough and breathlessness occurred in 30% cases. Central nervous system manifestations in the form of drowsiness and seizures were seen in 25% cases. Signs of consolidation were seen in 40% of cases. Thrombocytopenia was seen in 37.5% patients. All patients had elevation of SGOT and SGPT, while 52% patients had elevation of serum alkaline phosphatase. Acute renal failure was seen in 33% patients. 10.4% patients required mechanical ventilation and6.25% died of multiorgan failure.

## Conclusion

Scrub typhus needs to be considered as an important differential diagnosis in febrile patients with elevated liver enzymes. Complete response with doxycycline was observed in 93.75% cases. Early diagnosis and appropriate treatment will prevent morbidity and mortality.

